# Central and Peripheral Fatigue Evaluation during Physical Exercise in Athletic Horses by Means of Raman Spectroscopy

**DOI:** 10.3390/ani13132201

**Published:** 2023-07-05

**Authors:** Giuseppe Acri, Barbara Testagrossa, Giuseppe Piccione, Francesca Arfuso, Elisabetta Giudice, Claudia Giannetto

**Affiliations:** 1 Department of Biomedical, Dental and Morphological and Functional Imaging Sciences, University of Messina, Via Consolare Valeria, 98125 Messina, Italy; gacri@unime.it (G.A.); btestagrossa@unime.it (B.T.); 2 Department of Veterinary Sciences, University of Messina, Via Palatucci n 13, 98168 Messina, Italy; gpiccione@unime.it (G.P.); farfuso@unime.it (F.A.); egiudice@unime.it (E.G.)

**Keywords:** physical exercise, fatigue, horses, Raman spectroscopy

## Abstract

**Simple Summary:**

In athletic horses, the evaluation of the performance levels is of major importance during training to prevent sports injuries. Raman spectroscopy is an innovative technique that allows for a rapid evaluation of biological fluids. The application of Raman spectroscopy on serum samples collected from five Italian Saddle horses subjected to physical exercise confirms the utility of this technique for the assessment of the levels of serum metabolic biomarkers that cause the central and peripheral fatigue that occur in athletic horses during physical exercise. In particular, in two bands ((1300–1360) cm^−1^ and (1385–1520) cm^−1^), the metabolites involved in the insurgence of fatigue were identified. Lipids and tryptophan were identified in the (1300–1360) cm^−1^ band, and leucine, glycine, isoleucine, lactic acid, tripeptide, adenosine, and beta carotene were identified in the (1385–1520) cm^−1^ band. The area of each evaluated sub-band was used for evaluating changes in the conformational structure of proteins in sera that may depend on exercise. In conclusion, the application of Raman spectroscopy on blood serum samples represents a useful technique for secondary-structure protein identification to investigate the metabolic changes that occur in athletic horses during physical exercise.

**Abstract:**

The evaluation of the performance levels in athletic horses is of major importance to prevent sports injuries. Raman spectroscopy is an innovative technique that allows for a rapid evaluation of biomolecules in biological fluids. It also permits qualitative and quantitative sample analyses, which lead to the simultaneous determination of the components of the examined biological fluids. On the basis of this, the Raman spectroscopy technique was applied on serum samples collected from five Italian Saddle horses subjected to a standardized obstacle course preceded by a warm-up to evaluate the applicability of this technique for the assessment of central and peripheral fatigue in athletic horses. Blood samples were collected via jugular venipuncture in a vacutainer tube with a clot activator before exercise, immediately after exercise, and 30 min and 1 h after the end of the obstacle course. Observing the obtained Raman spectra, the major changes due to the experimental conditions appeared in the (1300–1360) cm^−1^ and (1385–1520) cm^−1^ bands. In the (1300–1360) cm^−1^ band, lipids and tryptophan were identified; in the (1385–1520) cm^−1^ band, leucine, glycine, isoleucine, lactic acid, tripeptide, adenosine, and beta carotene were identified. A significant effect of exercise was recorded on all the sub-bands. In particular, a change immediately after exercise versus before exercise was found. Moreover, the mean lactic concentration was positively correlated with the Raman area of the sub-band assigned to lactic acid. In this context, the application of Raman spectroscopy on blood serum samples represents a useful technique for secondary-structure protein identification to investigate the metabolic changes that occur in athletic horses during physical exercise.

## 1. Introduction

In physiology, fatigue is defined as the muscle incapacity to sustain and maintain its ability to produce work overtime. Fatigue is an important bodily defense system because it ensures that irreversible damage does not occur as a consequence of stresses imposed by physical activity [1]. In continuous and specific training sessions, a good athlete aims to delay its appearance to maintain high-performance levels [1]. There are many different fatigue classification methods. On the basis of its duration, fatigue is defined as acute if the physiological modification due to physical exercise can be restored after recovery, whereas if fatigue is not relieved by rest, then it is defined as chronic [2]. On the basis of its origin, fatigue is defined as peripheral if the incapacity to produce work is located in the neuromuscular junction, whereas it is defined as central if the central nervous system (CNS) reduces the neural drive to the muscle [2]. From a biochemical point of view, peripheral fatigue is due to the depletion of muscle glycogen and phosphocreatine (PCr) stored in the muscle [1]. In addition, it is caused by a marked decrease in the muscle pH due to the increase in the proton concentration following the accumulation of lactic acid [1]. During the early stage of moderate-intensity exercise, the primary muscle energy source is a carbohydrate, such as muscle glycogen or plasma glucose; when the exercise is continued and the carbohydrate is reduced both in muscle and in plasma, free fatty acids become a new energy source [3]. A low blood glucose concentration also mainly contributes to the central fatigue mechanism via the reduction in the glucose availability to the CNS [1,3]. The increase in brain serotonin is another determining factor for central fatigue insurgence. The increase in this molecule in the brain is due to an increase in its precursor (tryptophan) at this level. The transport of tryptophan to the brain is favored by the changes in the blood levels of many molecules involved in metabolic modifications due to exercise. In particular, the increased plasma levels of non-esterified fatty acids (NEFAs) induce an increase in the free tryptophan due to its displacement from its plasma binding site, albumin. Contextually, there is a decrease in the branched-chain amino acid (BCAA) levels due to an increase in the muscle uptake. Because the transport of tryptophan to the brain is influenced by competition with BCAAs for the use of the same membrane transporter, the increase in free tryptophan together with the decrease in BCAAs enhances the transport of tryptophan across the brain barrier, providing the substrate for serotonin synthesis, the mediator of the reduction in the motor drive [1,4].

In athletic horses, the assessment of the exercise capacity and training response is of major importance for animal welfare [5]. When athletic horses compete at levels above their exercise capacity, injuries can occur. In the last years, the principal physiological parameters, such as heart rate, blood lactic acid and glucose, respiratory parameters, maximum power output, and maximum oxygen uptake, have been monitored using new devices (heart-rate meters, lactate and glucose analyzers) [6]. All these techniques are non-invasive, but they do not provide information about the involved metabolic pathways. Understanding fatigue mechanisms may lead to the development of better training methods and the prevention of injuries. Recently, multivariate approaches to metabolomic analysis have been used to understand biological mechanisms [7]. With respect to biological endpoints, quantifications of metabolomes could elucidate biological phenomena in other omic studies, such as genomics, transcriptomics, and proteomics [8,9]. However, this approach is invasive because it requires a biopsy.

Raman spectroscopy, a technique for the analysis of biological fluids, which has been proven effective for the conformational analysis of proteins [10,11,12], was used in our study. Raman spectroscopy is widely used in the study of human biofluids (e.g., tears [13], urine [14], and sweat [15]).

Raman spectroscopy is commonly used in the unraveling of the molecule-specific spectral signatures of different biomolecules, including nucleic acids, lipids, carbohydrates, and complex biological systems, such as tissues, cells, etc., that are made up of such biomolecules [16]. It is broadly employed in the investigation of changes in the structure of protein aggregation and amyloid fibril formation [11,17]. Raman spectroscopy represents an inelastic light-scattering phenomenon that produces a vibrational spectrum of the specific molecule analyzed [18]. All biological systems are composed of biochemicals, such as lipids, proteins, nucleic acids, carbohydrates, etc. Vibrations from all the modes comprising the chemicals are manifested in the Raman spectra [19]. It represents an essential methodology in chemistry, physics, biology, material science, and medicine [20,21,22,23]. Recently, amino acids, which represent the main components of proteins, enzymes, bacteria, and viruses, have been successfully characterized by the use of Raman spectroscopy analysis [24]. The main advantage of applying Raman spectroscopy for the structural analysis of proteins is its water insensitivity, allowing for the characterization of biological materials in solution. Thereby, the biological activity of proteins is preserved, furnishing amino acid signature regions in the Raman signal [25].

Studies examining the metabolic effects of exercise in horses have focused on blood-related measures [26]. The challenge is represented by the choice of the appropriate biomarker to provide information about the focus of the study. In this context, Raman spectroscopy is a technique capable of analyzing complex molecular compositions based on the acquisition spectrum. By using Raman spectroscopy, due to the unique signatures of substances, countless biomarkers and metabolites can be analyzed in parallel within a few minutes, enabling a fast response [27]. Currently, laboratory diagnostics is based on individual chemical detection reactions, each requiring the sample volume, reagents, and complex infrastructure. In contrast, Raman spectroscopy represents a reagent-free technique that is easy to perform and provides abundant information about biomarkers in a single step.

The aim of the present prospective study was to evaluate the use of Raman spectroscopy to assess the modifications of the levels of serum metabolic biomarkers, collected before and after an obstacle course, that happen in athletic horses.

## 2. Materials and Methods

### 2.1. Horses and Sample Preparation

The research complied with the guidelines of Good Clinical Practices (EMEA, 2000) and the Italian and European regulations on animal welfare (Directive 2010/63/EU). According to national legislation, no ethics committee approval was needed for the study. All treatments, housing, and animal care reported were carried out following the standards recommended by the European Directive 2010/63/EU, section III, point (b). Five regularly trained gelding Italian Saddle horses aged between 8 and 10 years old, with a mean bodyweight of 510 ± 35 kg, were enrolled in this study. All horses had the same level of training, followed the same training program (six days per week), and performed competition in the same category (1.25 cm obstacle course). Clinical examination, routine hematology, and biochemistry analyses were performed under rest conditions, before starting the study, by a vet of the research team, who supervised the animals for the experimental period. Only healthy subjects were considered for the study. Each horse was enrolled after the signature of an owner’s informed consent. All the horses were housed and trained in the same horse-training center. All the horses were housed in individual boxes (3.50 × 3.50 m) and fed 11 ± 2 kg/day/horse of good-quality alfa-alfa hay (Meticago sativa) and 4.0 ± 0.5 kg/day concentrates (crude protein: 16%; crude fat: 6%; crude fiber: 7.35%; ash: 10.09%; sodium: 0.46%; lysine: 0.85%; methionine: 0.35%; omega-3: 0.65%), distributed in three meals (at 6:30 a.m., 12:00 a.m., and 7:30 p.m.). Water was available ad libitum. Horses were subjected to a standardized obstacle course preceded by a warm-up. The warm-up exercises consisted in walking (5 min), trotting (10 min), and galloping (5 min). Six jumps (3 verticals and 3 long jumps) of a 0.8–1.20 m height and 1.15 m width were also included in the warm-up (10 min). The exercise consisted of a 350 m long trail with eleven 1.25 m high jumps (5 vertical jumps, 5 long jumps, and 1 double-vertical and long jump). From each subject, four blood samples were collected via jugular venipuncture in 2 mL vacutainer tubes with clot activators (Terumo Corporation, Tokyo, Japan) before the exercise, immediately after the exercise, and 30 min and 1 h after the end of the obstacle course (t = 30 min and t = 60 min, respectively). The blood collection procedure is generally well tolerated in horses; an analgesic button is not necessary for this species [28]. Blood samples were centrifuged at 3000 rpm for 10 min, at room temperature, within 30 min from the collection. The obtained sera were stored for one week at −20 °C until Raman analysis. The rectal temperature (HI92704, Hanna Instruments, Bedfordshire, UK), heart rate (Polar S-610), blood lactic acid (Accusport, Boehringer, Mannheim, Germany), and glucose (Glucotrend, Roche Diagnostics, Basel, Switzerland) were recorded during the various data points of the experimental protocol to monitor the physiological responses of jumper horses to a simulated show-jumping course [29,30]. The values of the parameters obtained from blood sampling performed before the exercise represent the baseline values for each animal.

### 2.2. FT Raman Spectroscopy and Data Analysis

Raman spectra were obtained using a diode laser (785 nm wavelength) mounted on a DXR-SmartRaman Spectrometer (Thermo Fisher Scientific, Waltham, MA, USA). Data were acquired over the range of 400–3300 cm^−1^, with a maximum resolution of ~1.9 cm^−1^. The equivalent power was set to 24 mW, coming out from a 50 μm circular spot. Before Raman measurements, each sample was thawed by leaving it at room temperature for 20 min. Then, 250 µL of each horse’s serum sample was accommodated into a vial and covered by parafilm. The vials were accommodated in the sample holder, and the 180-degree sampling accessory for the DXR-SmartRaman Spectrometer was used for measurements. The laser target was focused on the bottoms of the vials. This region is not interested in “coffee ring effects”. To obtain high signal-to-noise ratio (S/R) spectra, an integration time for recording a Raman spectrum of 30 s and 32 scans for any spectrum was chosen.

To avoid damaging the samples, an extremely low laser power (24 mW) was used, and 16 min was necessary for a total acquisition, as previously reported for dog and human sera [12,23]. The protein degradation due to the temperature effect was excluded because no invalid reading of the spectra was obtained. The Raman spectrometer returned a single spectrum after 32 sample exposures. To obtain adequate information from acquired spectra, the spectrometer software (OMNIC for Dispersive Raman 9.1.24, Copyright© 1992–2012 Thermo Fisher Scientific Inc., Waltham, MA, USA) performed an automated background removal. After the acquisitions of Raman spectra, a manual baseline correction was executed using a spline algorithm, consisting in a cubic polynomial interpolation.

Once this cubic polynomial is fitted, the complete baseline is automatically subtracted from the original spectrum to yield the corrected one.

Each acquired spectrum was fitted by a multiple-curve-fitting routine built in the Omnic software (OMNIC for Dispersive Raman 9.1.24 software, Waltham, MA, USA). The used fitting procedure consisted in a nonlinear regression procedure (a nonlinear least-squares fitting) that adjusts the parameters in small steps to improve the goodness of the fit by minimizing the sum of the squares of the vertical deviations. Then, if the procedure converges, one can be safe that altering any of the parameters a little bit will make the fit worse.

The experimental data were deconvoluted using a Gaussian profile, with 10 cm^−1^ bandwidths. The software initiates the process of automatically adjusting the peak center height and width to produce a composite spectrum that matches the original. The convergence routine in Omnic is a Fletcher–Powell–McCormick algorithm. The convergence is determined by the ratio of the root mean square (rms) of the residual to the rms noise of the spectrum. For each fitting session, multiple iterations were performed to minimize the value of the F-Test and obtain the “best-fit” solution. The problem of finding a local (“false”) minimum was overcome by running each nonlinear regression several times (20 on average). The obtained F-Statistic values ranged between 0.001397 and 0.003379 (using a significance level of *p* = 0.05), confirming that there were no statistically significant differences between the original and composite spectra in all cases.

The second derivative was computed on the fitted curve of each original spectrum. The analysis of the second derivative of the spectra was applied to obtain a first indication of the minimum number of band components and their peak positions, according to procedures already successfully applied in Raman spectrum analysis [31], in which the minima in the obtained profiles represent the center frequencies of the sub-bands. The analysis of the second derivative was computed using OriginPro 9.0 software (OriginLab Corporation, Northampton, MA, USA).

One of the main advantages of the analysis of the n-th derivative, and, in particular, the second derivative, is that the objective possibility is performed without an arbitrary choice of the deconvoluted parameters. Quantitative analysis of the secondary structure of serum spectra assumes that a band of the spectrum can be considered as the linear sum of several fundamental elements of the secondary structure [32].

After the mathematical computation, two main sub-bands were determined in the (1300–1360) cm^−1^ band, centered at A ~1312 cm^−1^ and B ~1336 cm^−1^. In the (1385–1520) cm^−1^ band, seven main sub-bands were recognized, centered at 1~1393 cm^−1^; 2~1410 cm^−1^; 3~1437 cm^−1^; 4~1455 cm^−1^; 5~1470 cm^−1^; 6~1489 cm^−1^; and 7~1516 cm^−1^.

The area of each evaluated sub-band was used for evaluating changes in the conformational structure of the proteins in the sera.

In addition, another approach was considered, and the contribution of each sub-band area to the total one (Abiomarker/Aband), in percentage terms, was evaluated.

### 2.3. Statistical Analysis

Statistical analysis was performed using MATLAB^®^ R2018b (MathWorks Inc., Natick, MA, USA). The dependence of the sub-bands on different parameters was assessed by fitting a linear mixed-effects model (function “fitlme”), performed at different acquisition steps (before the exercise, immediately after the exercise, at t = 30 min after the end of the exercise, and at t = 60 min after the end of the training session), in the form of a categorical variable, as a fixed effect, and the subject, as a random effect. Groups before exercise, immediately after exercise, and at t = 30 min and t = 60 min were compared among them (Tukey post hoc comparison). The correlation coefficient (r) between the serum concentration and Raman area was determined. Regression lines and 95% confidence intervals for the different data recorded of each experimental condition were determined. The significance threshold for *p*-values was set to 0.05, and the results were expressed as means ± standard deviations (SDs).

## 3. Results

Table 1 shows the rectal temperature, heart rate, blood lactic acid, and glucose in the different data points of the experimental protocol.

**Figure 1:**
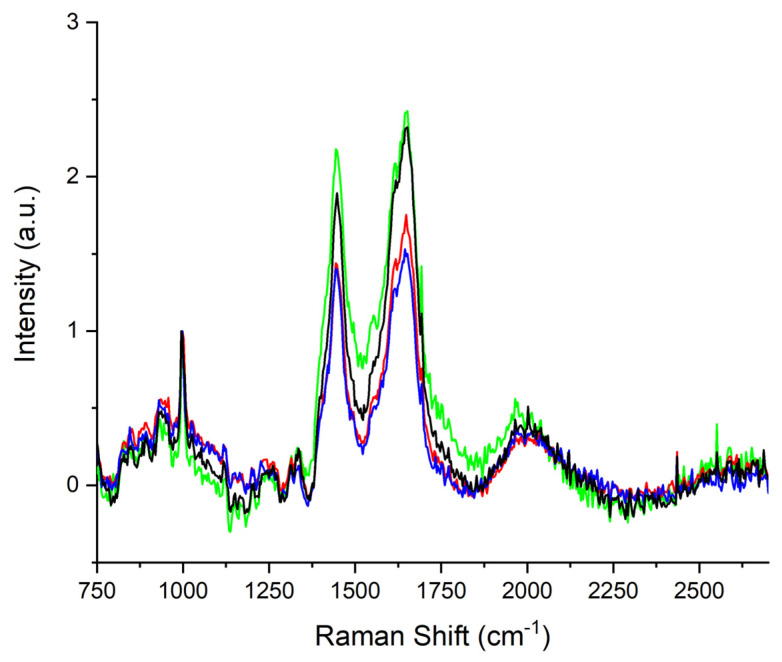
Average Raman spectrum of horses’ sera collected before exercise (black line), after exercise (blue line), 30 min after the end of exercise (red line), and 60 min after the training session (green line).

All the spectra reveal the typical protein vibrational modes. In particular, the phenylalanine (Phe) n-ring band located at ~1000 cm^−1^ was used as an internal standard to normalize the spectra, as it is insensitive to microenvironments. Figure 1 shows the normalized average Raman spectra of the horses’ sera collected during the experimental period.

**Figure 2:**
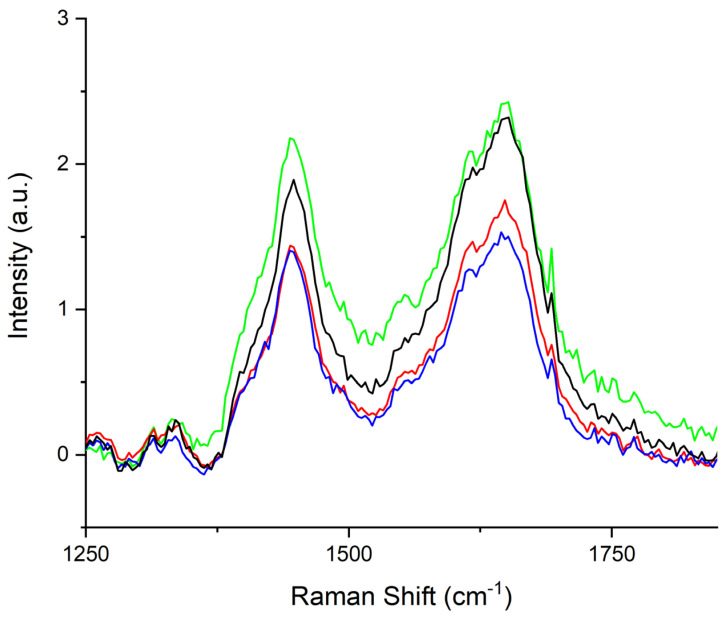
Average Raman spectrum, in the (1250–1800) cm^−1^ range, of horses’ sera collected before the training session (black line), immediately after exercise (blue line), 30 min after exercise (red line), and 60 min after exercise (green line).

The acquired spectra were visually similar, except for the large band detected in the (1250–1800) cm^−1^ range. In particular, differences in the (1300–1360) cm^−1^ and (1385–1520) cm^−1^ bands are evident. These differences are shown in Figure 2. The center frequencies of the sub-bands are correlated to metabolic biomarkers related to fatigue. We report the tentative assignment in Table 2 (1300–1360 cm^−1^) and Table 3 (1385–1520 cm^−1^).

**Figure 3:**
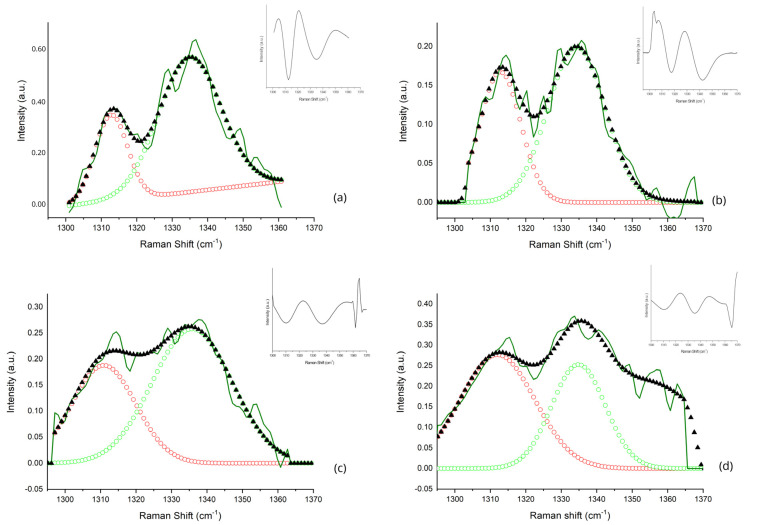
Curve-fitting results in the (1300–1360) cm^−1^ band: before the training session (**a**), immediately after exercise (**b**), 30 min after exercise (**c**), and 60 min after exercise (**d**). The dark-green line refers to the average experimental acquired spectrum; black triangles indicate the best fit of the average spectra. The detected sub-bands, obtained from deconvolution, are indicated. The sub-band centered at ~1312 cm^−1^ is indicated as red circles, whereas the sub-band centered at ~1336 cm^−1^ is showed as green circles, and their tentative assignment is reported in Table 2. In the upper-right corner of each graph, the second derivative of the fitted original spectra is reported, and the minima represent the center frequencies of the sub-bands.

The deconvoluted sub-bands in the (1300–1360) cm^−1^ region are reported in Figure 3a–d and refer to the different steps of the sample acquisition: (a) before exercise; (b) after exercise; (c) 30 min after the end of exercise; (d) 60 min after the end of the training session. After exercise, a decrease in the intensity of the bands was observed. Different contributions of the two sub-bands were observed in all data points. A total of 60 min of recovery was not enough to restore the band intensity and conformation observed at the basal data point.

**Figure 4:**
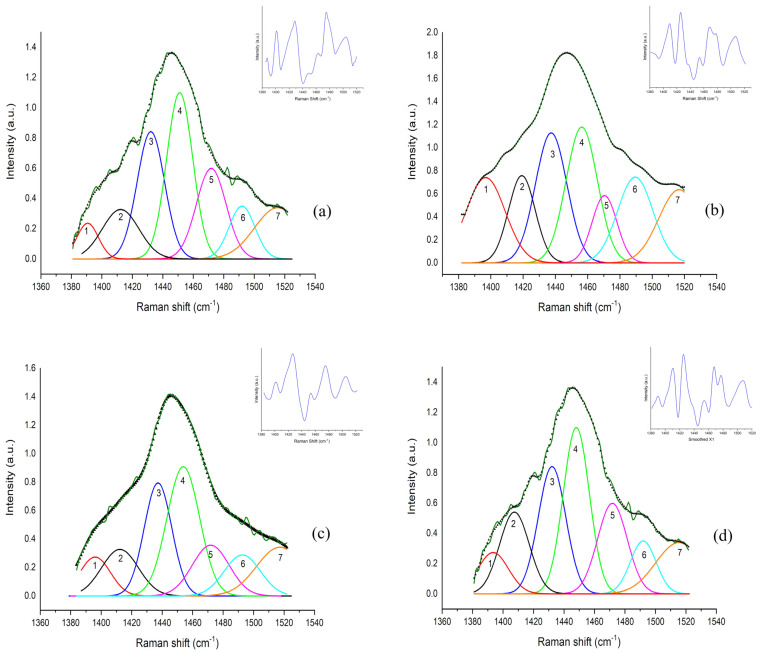
Curve-fitting results in the (1385–1520) cm^−1^ band: (**a**) before starting training session; (**b**) after exercise; (**c**) 30 min after the end of exercise; (**d**) 60 min after the training session. Dark-green line refers to average experimental acquired spectrum; black triangles indicate the best fit of the average spectra. The detected sub-bands, obtained from deconvolution, are enumerated and showed with the following colors: red (~1393 cm^−1^), black (~1413 cm^−1^), blue (~1437 cm^−1^), green (~1455 cm^−1^), purple (~1470 cm^−1^), cyan (~1489 cm^−1^) and orange (~1516 cm^−1^). The tentative assignment of all the detected sub-bands is reported in Table 3. In the upper-right corner of each graph, the second derivative of the fitted original spectra is reported, and the minima represent the center frequencies of the sub-bands.

The deconvoluted sub-bands in the (1385–1520) cm^−1^ region are reported in Figure 4a–d and refer to the different steps of the sample acquisition: (a) before exercise; (b) after exercise; (c) 30 min after the end of exercise; (d) 60 min after the end of the training session. After exercise, an increase in the intensity of the bands was observed. After exercise, different contributions of the seven sub-bands were observed with respect to the before-exercise data point. At 30 and 60 min after the end of exercise, the band intensity and conformation were similar to the before-exercise data point.

In Table 4 and Table 5, the Tukey’s post hoc significances are reported for the sub-band areas (mean values ± SDs) calculated in both the (1300–1360) cm^−1^ and (1385–1520) cm^−1^ ranges. In particular, sub-band A increased after exercise with respect to before, decreased 30 min after exercise with respect to after exercise, but did not reach the value at rest, and continued to decrease 60 min after the exercise, reaching the basal value. Sub-band B increased 30 min after exercise with respect to after exercise; 60 min after exercise, the recorded value was statistically lower than after 30 min, after exercise, and before. Sub-bands 1, 4, and 5 increased after exercise with respect to the basal value, and at 30 min after the end of exercise, started to decrease and continued decreasing 60 min after the end of exercise. Sub-bands 2 and 3 showed the opposite trend to sub-bands 1, 4, and 5. Sub-band 6 decreased after and 30 min after the end of exercise with respect to the basal value; and sub-band 7 decreased 60 min after the end of exercise with respect to after and 30 min after the end of exercise.

**Figure 5:**
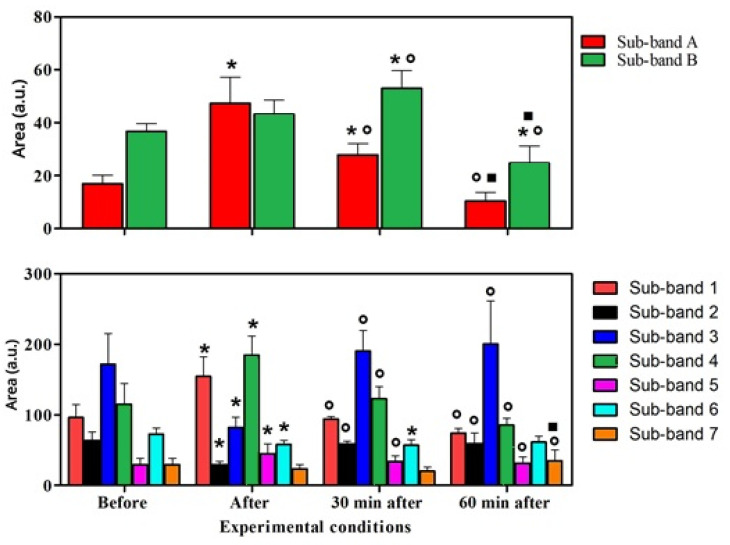
Trend of the area investigated inside the (1300–1360) cm^−1^ and (1385–1520) cm^−1^ ranges, expressed in arbitrary units, and the statistical differences due to the physical exercise. * vs. basal; ◦ vs. after; ▪ vs. 30 min after the end of exercise.

The analysis of the absolute-area values, depicted in Figure 5, revealed a significant effect of exercise on all the considered sub-bands. In particular, in the (1300–1360) cm^−1^ range, the A sub-band, assigned to collagen/lipids, statistically increased immediately after the exercise with respect to the basal value, and statistically decreased at t = 30 min, finally reaching the basal value at t = 60 min. The tryptophan (B sub-band) area statistically increased until t = 30 min with respect to the basal value and then returned to the basal value 60 min after the end of exercise. In Figure 5, the (1385–1520) cm^−1^ band is analyzed. All the sub-bands statistically changed immediately after the end of exercise, except for the beta-carotene sub-band (sub-band 7). The 1, 4, and 5 sub-bands, assigned to leucine, lactic acid, and tripeptide, respectively, statistically increased immediately after the end of exercise with respect to the basal value and gradually decreased at t = 30 min. On the contrary, the 2 and 6 sub-bands, assigned to glycine and adenosine, respectively, statistically decreased after the exercise. Both the absolute and percentage areas increased after the exercise and came back to the basal value at t = 30 min.

**Figure 6:**
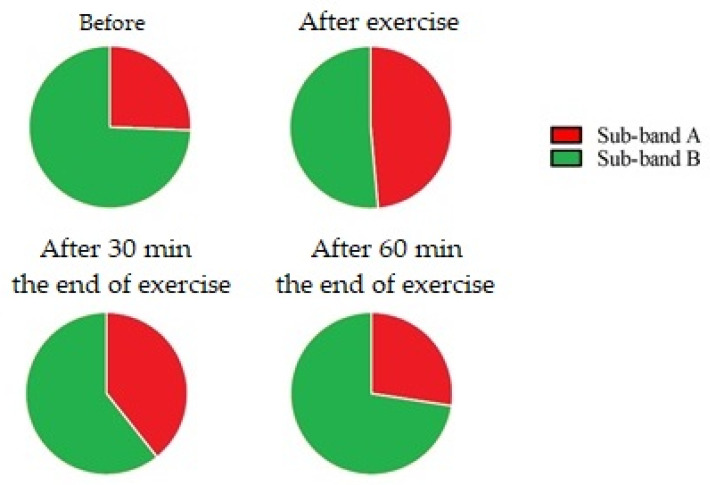
Percentages of sub-bands A (1312 cm^−1^—lipids) and B (1336 cm^−1^—tryptophan) found in the total area of the (1300–1360) cm^−1^ Raman band.

**Figure 7:**
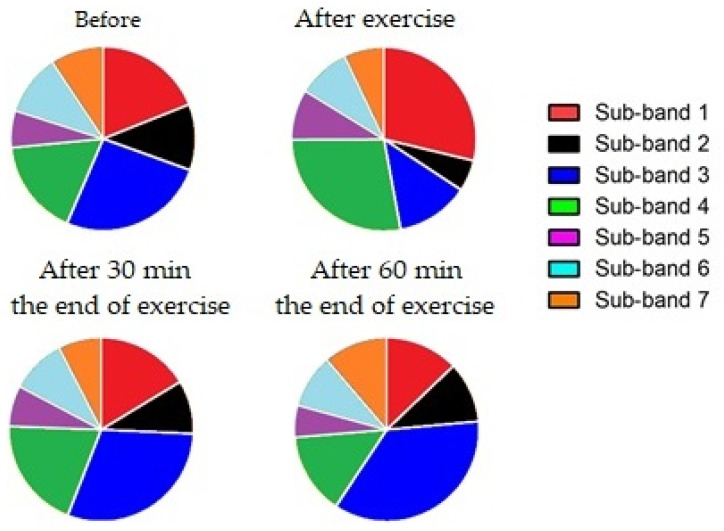
Percentages of sub-bands 1 (1393 cm^−1^—leucine), 2 (1413 cm^−1^—glycine), 3 (1347 cm^−1^—isoleucine), 4 (1455 cm^−1^—lactic acid, lipids), 5 (1470 cm^−1^—tripeptide), 6 (1489 cm^−1^—adenosine), and 7 (1516 cm^−1^—beta carotene) found in the total area of the (1385—1520) cm^−1^ Raman band.

When the percentage values of each sub-band in the (1300–1360) cm^−1^ range were considered, we noticed that in all experimental conditions, except for the results immediately after the exercise, where the A and B sub-bands equally contribute to the total area, the major contribution was due to the B sub-band (Figure 6). The B sub-band is attributed to the tryptophan biomarker, and its modification is the effect of the exercise and biochemical pathway that changes between an untrained and trained state. After physical exercise, the tryptophan decreased, and it increased 30 min after the end of the exercise. The percentage value returned to the initial conditions 60 min after the end of the workout. Within the (1385–1520) cm^−1^ range, the 1–4 sub-bands represent the largest changes (in percentage terms) in the total area (Figure 7).

Sub-band 1 is related to leucine, which plays a fundamental role in metabolic regulation. Leucine is essential to amplify the signal for protein synthesis and, soon after exercise, it suggests that the post-exercise consumption of amino acids stimulates the recovery of muscle protein synthesis via translation regulations. Sub-band 2 is associated with glycine, a non-essential amino acid in muscles, tendons, ligament, and skin collagen. Intense physical activity increases the need for glycine. Sub-band 3 is attributed to isoleucine, which participates in the process of muscle recovery after exercise. Sub-band 4, which refers to lactic acid, causes muscle fatigue and soreness.

**Figure 8:**
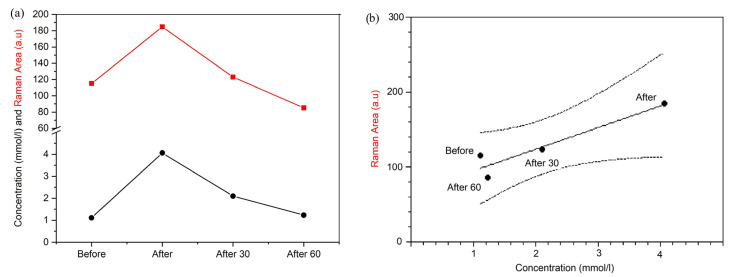
Comparison between the trend (**a**) of lactic acid concentration (mmol/L) obtained using the gold-standard methodology (black line) and Raman area (a.u.) of the sub-band centered at 1455 cm^−1^ (red line), together with the linear regression (**b**).

In order to demonstrate that Raman analysis could be a suitable tool for biomolecule identification and behavior, in Figure 8a, we compare the trend of the lactic acid concentration (mmol/L), reported in Table 1 and obtained using the gold-standard methodology (black line), and the Raman area (a.u.) of the sub-band assigned to lactic acid (red line). The mean lactic acid concentration was positively correlated with the Raman area of the sub-band assigned to lactic acid (*p* = 0.05; r = 0.94), as shown in Figure 8b.

## 4. Discussion

Raman spectroscopy represents a valid tool for the analysis of biological materials. The sharp bands that make up the Raman spectrum have distinct characteristics that are specific to each molecule. This allows for the easy identification of a specific substance thanks to its intrinsic biochemical difference, which makes it distinguishable from others [39].

We can identify metabolites by comparing the observed spectra to the reference ones. Band assignment is a proposal when comparing the bands obtained in the spectrum against a reference standard. The goodness of the obtained results is confirmed by comparing the sub-band center frequencies found in our study and the center frequencies of the peaks available in the literature and reported in Table 2 and Table 3. The area of the metabolite sub-bands is directly related to its abundance. As the abundance changes, the sub-band area belonging to the same metabolite varies, as confirmed in our study. In this study, we considered two different approaches to evaluate the behavior of the sub-bands resulting from Raman analysis: the analysis of the trend of the absolute-area values and the trend of the percentage areas of the sub-bands.

In the (1300–1360) cm^−1^ and (1385–1520) cm^−1^ ranges, serum constituents involved in the insurgence of fatigue have been found [44,45,46,48,49,50].

The trends of sub-bands assigned to a marker previously chosen as a candidate to explain the physiological alterations during exercise were compared with the serum concentrations of the corresponding metabolite. In the (1300–1360) cm^−1^ range, two sub-bands were identified, both with high relevance in exercise physiology. The two sub-bands were assigned to lipids (A) and tryptophan (B), respectively. Lipids are metabolites with a high energetic capacity that compete with glucose due to similar metabolic use. Several factors regulating energetic homeostasis influence the mobilization and use of lipids [51]. During aerobic exercise, lipids are an important energy source necessary for muscle contraction. The energy demand determines the liberation of free fatty acids from adipose tissue and their transport through the blood to the muscle [52]. Considering the absolute values of the detected areas, after exercise, the amount of lipids statistically increased with respect to the basal value, representing the response to physical exercise. At 30 min after the end of the training session, the lipid-area value decreased with respect to the value obtained after exercise. The value of the lipid area returned to values comparable to the basal ones 60 min after the end of the exercise. Tryptophan statistically increased at t = 30 min with respect to the basal and after-exercise values. Following exercise, rebound hyperinsulinemia has been observed [53], and insulin secretion has been known to induce a rise in blood tryptophan and a decrease in the number of amino acids in the blood [54], such as the suppression of the lipolytic rate [55]. At 60 min after the exercise, the area of the tryptophan sub-band statistically decreased, reaching a value lower than the basal one. The area trends of the A and B sub-bands, observed in the different data points, perfectly reflected the modification of the identified biomolecules (lipids and tryptophan) after a jumping course.

Differently, when we analyzed the behavior of the two metabolites in percentage area, we obtained a different trend. Before the exercise, 74.36% of the total area was represented by tryptophan and the remaining 25.64% by lipids (Figure 6). The behavior of tryptophan was characterized by a decrease immediately after the training session; then, it exhibited an increase until it returned to the initial values 60 min after the end of the training session. This behavior does not fit the trend observed when analyzing the absolute area that matches the results obtained in the blood sera in jumper horses, in which tryptophan increases after exercise [29]. Therefore, the obtained results revealed that the area percentage approach is not a suitable analysis tool for evaluating metabolic changes.

This is the first study that evaluates the use of Raman spectroscopy to assess the modifications of the levels of serum metabolic biomarkers. When the (1385–1520) cm^−1^ band was considered, we observed that, in all the seven analyzed sub-bands, the trend of the absolute-area values was similar to the percentage ones.

Despite the relatively slow average speed, show jumping is a strenuous discipline, as a large amount of energy is needed during the different phases of the jump (takeoff and landing) to overcome gravity [30]. During the jump, the anaerobic metabolism makes a significant contribution to the energy supply. The involvement of anaerobic metabolism during jumping exercises has been widely investigated [30,56], and has been demonstrated by the accumulation of lactic acid after the jumping course [56,57]. For this reason, we assigned to lactic acid the 4 sub-band detected in the (1385–1520) cm^−1^ range. Other peaks in the Raman spectra could be traced back to lactic acid (e.g., 750 cm^−1^; 830 cm^−1^; 930 cm^−1^ [58,59]), but they resulted in very weak intensity and were difficult to analyze. For this reason, we focused our attention on the sub-band centered at 1455 cm^−1^.

It is interesting how the Raman results of the lactic acid sub-band area and the concentration values of lactic acid reported in Table 1 have the same trend, as shown in Figure 8.

It is well known that the blood lactic acid concentration has significant variation before exercise and after racing and recovery in jumper horses [60]. Our results exactly reproduced the lactic acid variation due to the muscle mass recruitment during a jumper course. The increase in lactic acid, due to exercise, was followed by a rapid decline during the recovery phase as the consequence of the Cori cycle that uses the lactic acid released into the blood for gluconeogenesis in the liver. The resulting glucose is released and travels back to the muscle to restore the energy sources [56]. A similar trend was observed for the sub-band identified as leucine. It showed an increase after the end of the exercise. The leucine area reached the basal value 30 min after the exercise and continued to decrease 60 min after the training session.

A different trend was observed for the 2 and 3 sub-bands, assigned to glycine and isoleucine, respectively. Although it is well known that BCAAs are an important source of energy for muscle contraction, and their decrease has been correlated with fatigue insurgence, competing with tryptophan for the same transporter [61], the data reporting their blood levels after physical exercise are discordant and strictly linked to the muscle effort considered in horses [62]. The beta-carotene sub-band showed no statistically significant differences during the first three steps of our protocol, but, at t = 60 min, the area value statistically increased. This trend follows the beta-carotene behavior, involving the antioxidant processes of the body’s defense mechanism.

## 5. Conclusions

Raman spectroscopy analysis represents an applicable technique in the biomedical field that has been proven to be easy to perform, rapid, reproducible, and non-invasive. It does not require special chemical diagnostic kits, it is non-destructive, and the limit of the analysis is only represented by the degradation of the sample itself. Although the small sample size may have been warranted by the narrow inclusion criteria necessary to obtain a homogeneous sample of healthy horses with the same level of training, it is a limitation of the study. Our results can be considered a starting point for further studies on larger numbers of horses to confirm our results that made use of Raman spectroscopy on blood serum samples to investigate the metabolic changes in athletic horses that are involved in the insurgence of central and peripheral fatigue. It seems only right to emphasize that each amino acid spectrum is unique, and if it is exposed to a different environment because of conformational changes in the protein upon binding, this will appear in the Raman spectrum. The obtained results demonstrate that Raman spectroscopy analysis could be a suitable tool for biomolecule identification. Raman spectroscopy is very useful for protein identification, as well as for the analysis of their secondary structures. As this study demonstrated, this technique provides useful information for understanding horses’ responses to physical exercise, to monitor training programs, and to obtain an early poor-performance diagnosis, which can be performed in the field; in particular, the use of hand-held Raman and stimulated Raman scattering devices, operating at a microsecond acquisition speed, could improve the signal-to-noise ratio of acquired spectra by one order of magnitude [60], with the great advantage of biomolecule identification with respect to the high-wavenumber C-H window.

## Figures and Tables

**Table 1:** Rectal temperature, heart rate, blood lactate, and glucose in the different data points of the experimental protocol, indicating the physiological responses of jumper horses to a simulated show-jumping course.

Data Points				
	Before	After Exercise	After 30 min	After 60 min
Rectal temperature (°C)	36.98 ± 0.34	37.94 ± 0.43	37.56 ± 0.28	37.22 ± 0.19
Heart rate (bpm)	39.60 ± 2.60	199.00 ± 21.90	55.20 ± 3.34	40.80 ± 3.03
Lactic acid (mmol/L)	1.11 ± 0.07	4.06 ± 0.34	2.10 ± 0.66	1.23 ± 0.18
Glucose (mg/dL)	74.80 ± 6.76	55.60 ± 8.90	76.40 ± 4.33	76.20 ± 6.26

**Table 2:** Tentative assignment of the detected sub-bands in the Raman spectrum region (1300–1360) cm^−1^.

Sub-Band ID	Center Frequency (cm^−1^)	Metabolic Biomarker	Vibrational Mode	References
A	1312	Lipids	τ(CH_3_CH_2_)	[31,33,34]
B	1336	Tryptophan	γ(CH_2_)	[35,36]

τ, twisting mode; γ, wagging.

**Table 3:** Tentative assignment of the detected sub-bands in the Raman spectrum region (1385–1520) cm^−1^.

Sub-Band ID	Center Frequency (cm^−1^)	Metabolic Biomarker	Vibrational Mode	References
1	1393	Leucine	γ(CH_2_), δ(CCH)	[32,36,37]
2	1413	Glycine	s(COO^−^)	[36,38]
3	1437	Isoleucine	Cγ—asym rock; C δsym bend	[32,37,38,39]
4	1455	Lactic Acid, Lipids	CH_3_ asym bend; CH_3_ rock	[40,41,42,43,44,45]
5	1470	Tripeptide	σ(CH2); γ(NH_3_^+^)	[36]
6	1489	Adenosine	σ(CH2)	[46]
7	1516	Beta Carotene	C-C sym; s(C=C)	[47]

γ, wagging; δ, deformation; s, stretching; σ, scissoring; asym rock, asymmetric rocking; sym bend, symmetric bending.

**Table 4:** Mean values ± standard deviations expressed in arbitrary units of the sub-band area observed in the 1300–1360 cm^−1^ range in all experimental data points together with their statistical differences (Tukey’s post hoc comparison test). NS, not significant.

1300–1360 cm^−1^ Range				
Sub-band A (Collagen/lipids)				
Data points				
	Before	After exercise	After 30 min	After 60 min
Mean ± SD	16.92 ± 3.26	47.37 ± 9.87	25.81 ± 14.35	10.46 ± 3.21
Before		0.0001	0.01	NS
After exercise	0.0001		0.001	0.0001
After 30 min	0.01	0.001		0.001
After 60 min	NS	0.0001	0.001	
**Sub-band B (Tryptophan)**				
**Data points**				
	**Before**	**After exercise**	**After 30 min**	**After 60 min**
Mean ± SD	36.77 ± 2.86	43.16 ± 5.40	52.98 ± 6.63	24.74 ± 6.50
Before		NS	0.001	0.01
After exercise	NS		0.01	0.0001
After 30 min	0.001	0.01		0.0001
After 60 min	0.01	0.0001	0.0001	

**Table 5:** Mean values ± standard deviations expressed in arbitrary units of the sub-band area observed in the 1385–1520 cm^−1^ range in all experimental data points together with their statistical differences (Tukey’s post hoc comparison test). NS, not significant.

	1385–1520 cm^−1^ Range				
	Data Points				
		Before	After Exercise	After 30 min	After 60 min
Sub-band 1 (Leucine)	Mean ± SD	96.38 ± 18.61	154.70 ± 27.84	94.06 ± 3.56	74.43 ± 0.71
	Before		0.0001	NS	NS
	After exercise	0.0001		0.0001	0.0001
	After 30 min	NS	0.0001		NS
	After 60 min	NS	0.0001	NS	
Sub-band 2 (Glycine)	Mean ± SD	63.68 ± 12.28	29.26 ± 5.02	58.90 ± 4.24	59.46 ± 14.94
	Before		0.001	NS	NS
	After exercise	0.001		0.01	0.001
	After 30 min	NS	0.01		NS
	After 60 min	NS	0.001	NS	
Sub-band 3 (Isoleucine)	Mean ± SD	171.80 ± 43.51	81.80 ± 15.08	190.50 ± 29.00	200.70 ± 60.85
	Before		0.01	NS	NS
	After exercise	0.01		0.001	0.001
	After 30 min	NS	0.001		NS
	After 60 min	NS	0.001	NS	
Sub-band 4 (Lactic acid)	Mean ± SD	115.10 ± 29.62	184.80 ± 27.36	123.10 ± 16.79	85.34 ± 9.97
	Before		0.001	NS	NS
	After exercise	0.001		0.01	0.001
	After 30 min	NS	0.01		NS
	After 60 min	NS	0.001	NS	
Sub-band 5 (Tripeptide)	Mean ± SD	29.48 ± 9.15	44.70 ± 14.39	34.16 ± 7.98	31.72 ± 8.91
	Before		0.001	NS	NS
	After exercise	0.001		0.01	0.001
	After 30 min	NS	0.01		NS
	After 60 min	NS	0.001	NS	
Sub-band 6 (Adenosine)	Mean ± SD	73.93 ± 8.36	58.24 ± 5.66	57.16 ± 8.02	61.68 ± 8.49
	Before		0.01	0.01	NS
	After exercise	0.01		NS	NS
	After 30 min	0.01	NS		NS
	After 60 min	NS	NS	NS	
Sub-band 7 (Beta carotene)	Mean ± SD	29.61 ± 9.08	23.35 ± 6.48	20.28 ± 5.91	35.05 ± 15.42
	Before		NS	NS	NS
	After exercise	NS		NS	0.05
	After 30 min	NS	NS		0.01
	After 60 min	NS	0.05	0.01	

## Data Availability

The data presented in this study are available on request from the corresponding author.
